# Editorial: Identification of novel biomarkers for retinopathy of prematurity in preterm infants by use of innovative technologies and artificial intelligence

**DOI:** 10.3389/fped.2024.1382858

**Published:** 2024-03-06

**Authors:** Carina Slidsborg, Alistair Fielder, M. Elizabeth Hartnett

**Affiliations:** ^1^ Department of Ophthalmology, Copenhagen University Hospital, Rigshospitalet, Glostrup, Denmark; ^2^ Division of Optometry & Visual Science, City, University of London, London, United Kingdom; ^3^ Department of Ophthalmology, Byers Eye Institute at Stanford University, Palo Alto, CA, United States

**Keywords:** prematurity, retinopathy of prematurity (ROP), biomarker discovery, innovative technologies, artificial intelligence, risk stratification, predictive factors, diagnosis

**Editorial on the Research Topic** Identification of novel biomarkers for retinopathy of prematurity in preterm infants by use of innovative technologies and artificial intelligence

Advances in our understanding of retinopathy of prematurity (ROP) have improved its management and outcomes (1–12). However, additional research is needed, including our ability to predict those infants at greatest risk of blindness. This special issue provides insight into new methods for identifying predictive and diagnostic factors, and future research that will refine our approaches.

ROP screening protocols have been implemented worldwide and differ based on the different ROP risk profiles (13, 14). Despite the differences in ROP progression and risk profiles, there is a recognized need to identify predictive and diagnostic biomarkers. Biomarkers should be interpreted as clinical characteristics, along with ocular signs and disease-related molecular expressions in fluids or tissues. New biomarkers can be used to predict ROP risk, refine ROP risk stratification, and potentially lead to treatment strategies tailored to the individual neonate. Innovative technologies, including machine learning and artificial intelligence (AI) tools and imaging methods using deep learning (DL) algorithms, have been used to better define the fundus features of the International Classification of ROP (ICROP), which has been the gold standard (15–17). These methods have been proposed to reach the level of human decision-making and to detect retinal characteristics that would otherwise go unnoticed during clinical examination (18–20).

In this research topic collection, we showcase the development and validation of the most recent DL ROP model by Rao et al. which successfully separated Indian neonates into no ROP and ROP from a large dataset of 227,326 anonymized wide-field fundus images from various camera types. Such innovative initiatives are especially important in countries with limited healthcare resources. For any novel AI tool, it should be kept in mind that model accuracy is the single most important element to ensure before clinical use. Common pitfalls include the application of small, skewed datasets, poor data quality, narrow equipment use, population/selection bias, and ethnic, regulatory, and legal issues. Importantly, AI tools also need to be repeatedly evaluated after use in routine clinical practice.

Recently developed handheld non-invasive optical coherence tomography (OCT) has led to the detection of new retinal and vitreous biomarkers in neonates. In this research topic collection, Mangalesh and Toth and Kubsad et al. summarized recent studies using handheld OCT imaging technology to detect biomarkers in neonates. Mangalesh and Toth described new ROP signs like thinning or splitting of the inner retinal layers, neovascular buds, pre-retinal neovascularization, and hyperreflective spots in both the retina and vitreous. In addition, Kubsad et al. highlighted punctate hyperreflective vitreous opacities and bands, cystoid macular edema, foveal deformation, and a thinner choroid. An interesting next step in the advancement of pediatric imaging technology is handheld, non-invasive wide-field OCT angiography, which may provide interesting ways to quantify areas of choroidal and retinal neovascularization, perhaps as indicators of disease activity.

Several studies have pioneered ROP biomarker research in the blood of preterm infants. Many have focused on the role that vascular endothelial growth factor (VEGF) and insulin-like growth factor (IGF-1) play in neovascular development in the neonatal retina (5, 11, 21–28). More recently, advancements have been made in high-throughput omics techniques that allow the exploration of molecular profiles of metabolites, proteins, lipids, gene variants, and micro-RNA. These OMIC studies aim to identify biological pathways regulated in ROP and upstream regulators of several signaling mechanisms important in the pathogenesis of ROP (29, 30). Recently, the application of customized panels to target specific proteins in these biological processes has become popular. In this research topic collection, Ling et al. used a prospective panel study to investigate serum levels of 40 inflammatory cytokines associated with ROP in neonates. The authors detected independent and significant regulation of some of these blood biomarkers in zone 1 ROP and stage 3 ROP, along with postmenstrual age, respiratory distress syndrome, necrotizing enterocolitis, and sepsis. Further studies in external large-scale datasets are needed to validate their findings and develop new experimental designs with insight into refined hypotheses.

Another important aspect of biomarker discovery is the acquisition of big data from various data sources such as registries, databases, and patient records, along with data management and bioinformatics. Here, Xie et al. presented an innovative approach to drug discovery from big data sources to unravel safe drug potentials for the treatment of ROP. The authors searched ROP genes and related pharmacologically safe molecules from the major established databases, PubMed and NCBI, and used bioinformatics tools to identify safe choices for future treatments.

The guest editorial team anticipates that advancements in innovative technologies, AI tools, and big data will advance future understanding of the pathophysiology of ROP at the individual infant level, leading to personalized ROP management. The success of this development will largely depend on a multidisciplinary collaborative approach between clinicians, researchers, data scientists, and technology developers. Therefore, we encourage a strong collaborative engagement of experts in this expanding field of research and hope that policymakers and foundations will support these endeavors. Most importantly, the success of these efforts will hopefully lead to lifelong improved vision and quality of life for many children around the world.
